# A Metabolic Index of Ischemic Injury for Perfusion-Recovery of Cadaveric Rat Livers

**DOI:** 10.1371/journal.pone.0028518

**Published:** 2011-12-14

**Authors:** Sinem Perk, Maria-Louisa Izamis, Herman Tolboom, Basak Uygun, Francois Berthiaume, Martin L. Yarmush, Korkut Uygun

**Affiliations:** 1 Center for Engineering in Medicine, Harvard Medical School, Massachusetts General Hospital, and the Shriners Hospitals for Children, Boston, Massachusetts, United States of America; 2 Division of Cardiac and Vascular Surgery, University Hospital Zurich, Zurich, Switzerland; 3 Department of Biomedical Engineering, Rutgers University, Piscataway, New Jersey, United States of America; Rutgers University, United States of America

## Abstract

With over 110,000 patients waiting for organ transplantation, the current crisis in organ transplantation is based on a lack of donors after brain-death (DBD). A very large alternative pool of donor organs that remain untapped are the donors after cardiac death (DCD), recovered after cardiac activity has ceased and therefore sustained some ischemic injury. Machine perfusion has been proposed as a novel modality of organ preservation and treatment to render such cadaveric organs, and in particular livers, transplantable. Two key issues that remain unaddressed are how to assess whether a DCD liver is damaged beyond repair, and whether machine perfusion has rendered an injured organ sufficiently viable for transplantation. In this work, we present a metabolic analysis of the transient responses of cadaveric rat livers during normothermic machine perfusion (NMP), and develop an index of ischemia that enables evaluation of the organ ischemic injury level. Further, we perform a discriminant analysis to construct a classification algorithm with >0.98 specificity to identify whether a given perfused liver is ischemic or fresh, in effect a precursor for an index of transplantability and a basis for the use of statistical process control measures for automated feedback control of treatment of ischemic injury in DCD livers. The analyses yield an index based on squared prediction error (SPE) as log(SPE) >1.35 indicating ischemia. The differences between metabolic functions of fresh and ischemic livers during perfusion are outlined and the metabolites that varied significantly for ischemic livers are identified as ornithine, arginine, albumin and tyrosine.

## Introduction

About 110,000 patients are currently on the organ transplant waiting list in the US, with the number increasing by 5% every year (United Network for Organ Sharing, www.unos.org, as of July 2011). The major untapped pool of donor organs that could be used to alleviate this crisis in organ transplantation are the organs obtained from Donors after Cardiac Death (DCD) [1]. For the liver, which this work focuses on, an estimated 4,000 waitlisted patients perish every year due to a lack of transplantable organs [2], while the estimated pool of DCD livers with ischemic time <60 mins is on the order of 6,000 grafts per year [3].

In the absence of cardiac output, ischemic damage increases in severity as a function of time. Beyond a certain cutoff (about 15 minutes for the heart and 30 minutes for the liver) graft survival in the recipient falls dramatically [4]. Preclinical studies with extracorporeal machine perfusion systems in porcine and murine models of DCD livers [5], [6], [7], [8], [9], [10], [11], including from our group [12], [13], [14], indicate that up to 60 minutes of warm ischemic damage can be successfully reversed, whereas static cold storage in preservation medium, the current clinical gold standard, simply exacerbates the damage and recipient animals do not survive. Research in machine perfusion systems is subsequently a very active field in donor organ recovery and preservation.

In humans however, cardiac death frequently occurs in uncontrolled environments (uDCD). Without objective metrics of ischemic duration and organ viability, uDCD organs cannot be safely transplanted. Another benefit of machine perfusion is that data can easily be procured and analyzed, providing those necessary metrics that describe organ status. Normothermic (37°C) Machine Perfusion (NMP) in particular allows the organ to be metabolically active producing measurable changes in perfusate metabolite composition that are comparable to its in vivo counterpart.

Since hepatic metabolism is a highly integrated network which features many metabolites that are auto- and cross-correlated in time, univariate techniques (such as ANOVA) which ignore the correlation structure between the metabolites and assume that these variables are independent of each other are inadequate to handle the problem complexity [15]. A suitable framework for developing an index of ischemia, and more broadly quantitative methods of organ quality control, is multivariate statistical process monitoring (SPM) methodologies [15], [16]. Multivariate SPM techniques can look at the whole picture to identify commonalities between different perfusions, correlations among variables as a function of ischemia, and trends in time.

The aim of this work is the development of an index of ischemia to evaluate DCD liver injury based on the organ's dynamic metabolic activity during machine perfusion. To create such an index, we first construct a multi-way principal component analysis (MPCA) liver perfusion model that captures the correlation structure between the metabolites during perfusion of fresh livers that were later successfully transplanted with >1 month survival, and defines the multivariate confidence limits for the metabolite concentrations in the perfusion medium. Quantitative evaluation of the process of recovery from ischemic injury by NMP is then done on an online basis, enabling the perfusionist to apply the necessary interventions that will optimize recovery. This is done by projecting the metabolite concentrations for the damaged livers onto the fresh liver MPCA model, revealing the differences in metabolic functioning of DCD livers compared to fresh livers. We then quantify the similarity of ischemic livers to fresh livers by employing the Squared Prediction Error (SPE) statistic, hence constructing an index of ischemia.

Further, we introduce an online multi-way partial least squares discriminant analysis (MPLSDA) model to predict the end-of-perfusion quality (i.e. ischemic or not) of a perfused liver during perfusion. This analysis complements the ischemia index by creating a means of classifying any given liver definitively as ischemic or fresh, and it is proposed as the basis for a future decision criterion of DCD liver transplantability.

## Materials and Methods

### Animals

Male Lewis Rats weighing 200–300 g were obtained from Charles River Laboratories (Wilmington, MA) and maintained in accordance with National Research Council guidelines. The Subcommittee on Research Animal Care, Committee on Research at the Massachusetts General Hospital, approved the experimental protocols. All animals were allowed to acclimatize for at least 2 days prior to any experimentation. Procured livers were either Fresh (F; n = 11) or exposed to one hour of warm ischemia (WI; n = 7) before they were placed in the perfusion system.

### Warm ischemia

Full details of the liver isolation protocol for donor livers are explained elsewhere [14]. WI was induced by placing livers in a temperature-controlled chamber filled with saline and maintained at 34±0.1°C for 1 hr during which time the portal vein and vena cava were cuffed. Ex vivo ischemia ensured a constant temperature [17] and enabled a severe model of warm ischemia [18]. After an hour of warm ischemia, livers to be machine-perfused were flushed with saline and then connected to the perfusion system. Fresh livers for reperfusion were flushed through the PV with 10 mls of saline upon clamping the vein in situ and were then placed in a bowl of room temperature saline to be cuffed at the PV and IVC; average warm ischemic time prior to reperfusion was 10±2 minutes.

### Normothermic Liver Perfusion

The perfusion medium contained phenol red-free Williams Medium E (WE, Sigma Chemical, St. Louis, MO). WE was supplemented with 2 u/L insulin (28.85 units/mg Humulin, Eli Lily, Indianapolis, IN), 100,000 u/L penicillin, 100 mg/L streptomycin sulfate (Gibco, Invitrogen, Grand Island, NY), 0.292 g/L L-glutamine (Gibco), 10 mg/L hydrocortisone (Solu-Cortef, Pharmacia & Upjohn, Kalamazoo, MI), and 1000 u/L heparin (APP, Schaumburg, IL). The primary circuit of the perfusion system comprised perfusion medium (perfusate) that recirculated by means of a peristaltic pump through a jacketed perfusion chamber, a membrane oxygenator, a heat exchanger, and a bubble trap. The oxygenator was gassed with a mixture of 74%N_2_/21%O_2_/5%CO_2_ and 100% O_2_ to maintain a constant pH. Fresh rat plasma (25% v/v) and erythrocytes (18–20% v/v) were collected earlier and added to the perfusate. The total perfusate volume was 55 to 60 mL. Perfusate hematocrit was sustained, nutrients were replenished, and metabolism by-products were diluted through dialysis. A hollow fiber dialyzer with a 2200 cm^2^ membrane area and a 30 kDa nominal molecular cutoff weight (SpectrumLabs, Rancho Dominguez, CA) enabled counter-current mixing of perfusate in the primary circuit with a reservoir of WE (dialysate) in a secondary circuit. Temperature within the system was maintained at 37°C.

Upon completion of cuffing of fresh livers (∼5 min) and after the period of warm ischemia for WI livers, they were immersed in perfusate in the perfusion chamber. Livers were perfused at a constant flow rate through the portal vein while maintaining portal pressure between 10 and 12 cmH_2_O. The effluent flowed freely from the SHVC and IVC into the surrounding medium. When the recipient hepatectomy was prepared, the liver was disconnected from the circuit, rinsed in a bowl of saline at room temperature, and weighed again before transplantation.

### Transplantation

A modification of the cuff technique designed by Kamada and Calne [19], [20], [21] was implemented and is described in detail elsewhere [14]. The anhepatic phase of the procedure was typically 13–15 minutes and did not exceed 17 minutes. Animals were hydrated with 8 ml/kg of warm (37°C) lactated Ringer's solution with 5% dextrose and 2 ml/kg of NaHCO_3_ 7%w/v (Abbott, North Chicago, IL) by penile vein injection.

The animals were put singly in clean cages, allowed to recover from anesthesia under an infrared lamp for half an hour, and subsequently returned to regular housing. During the first 12 hours post-transplantation animals were checked every 2 hours and subsequently every 8 hours for one week, and daily afterwards. All of the livers were successfully transplanted with >1 month survival rate.

### Metabolite Sampling

Perfusate and dialysate samples (1 mL) were collected hourly from the liver effluent and reservoir, respectively. Perfusate samples were first spun down at 3000 g before storing the supernatant at −80°C. Urea was assayed by reaction with diacetyl monoxime using a commercial assay kit (BUN, Sigma-Aldrich, St. Louis, MO). Ketone bodies were measured enzymatically, by following the appearance of NADH in the conversion to acetoacetate and the disappearance of NADH in the conversion to β-hydroxybutyrate in the presence of β-hydroxybutyrate dehydrogenase. Eighteen of the common amino acids (except tryptophan and cysteine) and ammonia were fluorescently labeled using the AccQ - Tag system (Waters Co., Milford, MA), separated by high-performance liquid chromatography (HPLC; Model 2690, Waters Co.) and quantified by a fluorescence detector (Model 474, Waters Co.) as previously described [22]. Lactate was measured using the enzymatic conversion to pyruvate and hydrogen peroxide with lactate oxidase from a commercially available kit (Trinity Biotech, Berkeley Heights, NJ). Albumin concentration was determined by an enzyme-linked immunosorbent assay using a polyclonal antibody to rat albumin [23]. Note that the dialyzer molecular cutoff weight was determined so that albumin could not pass through, and subsequently did not appear in the secondary dialysate circuit. Glucose measurements were quantified with an enzymatic assay kit through conversion to 6-phospo-gluconate (Glucose assay kit, Sigma).

### Statistical Preprocessing

Data consisted of 25 metabolites measured hourly for each perfusion (Table 1). 11 Fresh livers and 7 WI livers were perfused in total. During the initial data preprocessing obvious outliers (e.g. negative concentrations) were eliminated and replaced by the mean trajectory estimates [15]. The data sizes that are used for the statistical analysis is 11×25×6 for fresh livers and 7×25×6 for damaged livers. All variables (i.e. the metabolite concentrations) were mean-centered and unit-variance scaled for further statistical analyses.

**Table 1:** List of metabolites used in data analysis.

1	ACAC	10	Glutamine	19	Phenylalanine
2	Alanine	11	Glycine	20	Proline
3	Albumin	12	Histidine	21	Serine
4	Ammonia	13	Isoleucine	22	Threonine
5	Arginine	14	Lactate	23	Tyrosine
6	Asparagine	15	Leucine	24	Urea
7	Aspartate	16	Lysine	25	Valine
8	Glucose	17	Methionine		
9	Glutamate	18	Ornithine		

### Multi-way Principal Component Analysis (MPCA)

While the differences in metabolic activity between fresh and ischemic livers can be compared on a per-metabolite (i.e. univariate) basis, since the liver is very capable of converting these metabolites into each other such an analysis would have problems with identifying that a large number of small differences could also mean a significant alteration in an entire metabolic pathway; for instance a significant alteration in urea cycle can easily come through a combination of changes in citrulline, ornithine, urea, and arginine concentrations in perfusion media with individual p-values that are not significant, but the overall fluxes over the entire pathway significantly altered; hence a univariate analysis such as ANOVA or t-test would lead to a Type II error, i.e. a missed alarm or false negative. Alternatively a difference in a single metabolite could be falsely interpreted to mean significant alteration in an entire pathway, which is a Type I error (false alarm or false positive). In other words, evaluation of a highly interconnected network such as the hepatic metabolism based on a univariate analysis that does not account for the cross-correlations between variables is expected to lead to a significant amount of Type I and Type II errors.

Multivariate methodologies such as principal component analysis (PCA) have been proposed for the analysis of such datasets with many correlated variables [24], [25]. PCA captures the correlation structure between the variables and forms a model plane with fewer dimensions which explain the largest variations in the data, in effect distilling the many correlated variables (such as metabolites in a pathway) into a few variables that are uncorrelated with each other.

Briefly, PCA captures the correlation structure between the variables in **X** [(I×J), j = 1,…, J variables; i = 1,…,I independent samples] and forms a model plane with fewer dimensions (R principal component (PC) directions) using only the R largest variance directions. R is chosen such that adding additional components to the model does not provide additional significant information. Instead of working with highly correlated collinear variables (**X**), PCA yields fewer and uncorrelated projections (scores (**T**)).
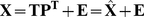
(1)


PCA is performed by singular value decomposition in the covariance of **X** and the loadings **P** (J×R) are derived. The R eigenvectors are the R highest variance directions. Scores, **T** (I×R), are the new uncorrelated variable projections onto the newly derived PCA plane. **E** is the residual matrix. Score biplots can then be used to reveal how similar *I* independent samples are to each other, reveal possible clustering among the samples and also used in determination of outlier samples. Loading plots show the correlation among *J* variables and identifies the variables that have high influence on each model score vector **t**.

**Figure 1:**
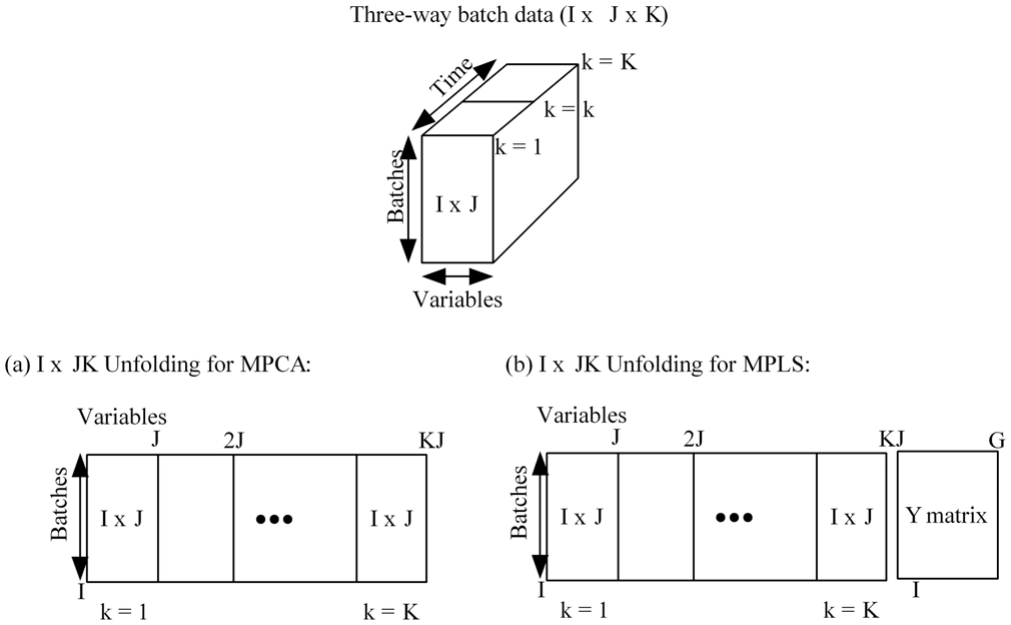
Unfolding of the (I×J×K) dynamic batch process data for MPCA and MPLS analyses.

For Statistical Process Monitoring (SPM) of dynamic datasets, PCA has been extended to multiway-PCA (MPCA). Multiway-PCA is used for the analysis of three dimensional data arrays **X** (I×J×K) where I independent processes are referred to as batches (i = 1,…, I). A multi-way PCA model is equivalent to an ordinary PCA model performed on a 2D matrix constructed by unfolding the three-way data array [15], [26], [27], [28], [29], [30]. An (I×J×K) data array can be unfolded by preserving the batch direction I and augmenting the J variable measurements taken at each time point *k* (k = 1,…, K) side by side resulting in an I×JK matrix, or by preserving the variable direction *J* and augmenting the variable trajectories for different batches resulting in a IK×J matrix (Figure 1). The former unfolding direction is the best to use in the SPM of batch processes since it considers the batch-to-batch variations and determines the ranges of variable trajectories during the batch. The latter unfolding technique provides information on the extent of the progress of a batch and can be used to determine the completion time of a batch [31].

For the statistical modeling of fresh liver perfusions with MPCA, an MPCA model was built using the remaining fresh liver samples and the confidence intervals around the model plane were determined. Later, the liver samples that were exposed to 1 hr warm ischemia (WI) were projected onto the fresh liver model plane, and new scores and residuals were calculated using:
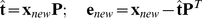
(2)
**P** loadings (size (JK×R) for batch processes) are used to calculate the new scores **t** (1×R) using the new batch trajectory **x**_new_ (1×JK).

#### Squared Prediction Error (SPE)

There are several measures to quantify similarity between two batch operations with multiple measurements at each time point, and evaluate if they are within normal operational bounds (also termed confidence limits). Multivariate statistics such as the Squared Prediction Error (also called Q-statistic) are used to determine the state of the batch progression and detect possible deviations in the new batches (**x**_new_, (1×JK)) from the reference batches [15], [26], [27], [28], [32].

SPE, in particular, captures the large deviations from the normal operating (NO) process that are not explained by the model and is calculated for the new batch using the residuals **e**_new_ [29],
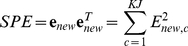
(3)


SPE statistic considers the residual space and determines the variation of a sample that is not explained by the model, hence, a large SPE statistic indicates that the observation under consideration contains a different correlation structure and is not explained by the model. For instance, if ischemic livers contain a correlation structure that is not captured by the fresh liver model, it will be indicated by a large SPE value. As such, SPE effectively distills a single quantitative variable from a large number of highly-correlated metabolite profiles, and comparison of a perfused liver to a set of ideal-condition livers can be accomplished simply by comparing the SPE values to each other.

An offline analysis of the metabolite trajectories (i.e. analysis at the end of a batch when data for all time points is ready) such as the SPE statistic determines the events that have taken place during the process. An online analysis (i.e. during perfusion) is more desirable since it provides information on the events taking place as the perfusion progresses and an estimate of the liver viability based on the current conditions. Accordingly, online SPM enables regulation of the process (e.g. adjusting the operational parameters such as oxygenation, flow rate etc.) to improve the final organ viability.

For online analysis each independent score is calculated at time k using,
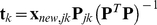
(4)where 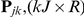 are the model loadings until time interval k. Residuals are then calculated using 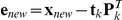.

For such an online analysis, SPE statistic can be calculated at each time point k (k = 1 … K) for the new batch as:
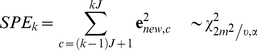
(5)

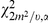
 is the critical value of the chi-squared variable with 2 m^2^/υ degrees of freedom at significance level α m and υ are the sample mean (m) and variance (υ) of the SPE sample at each time k [28].

Contribution plots can be used as a diagnostic tool to identify the variables that are contributing to the deviation. The variable contributions to SPE statistic for batch *i* are calculated using [33],
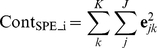
(6)


In order to quantify the total variation of each variable in the residual space, contributions to the normalized error vector are calculated as [34],
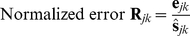
(7)where **e**_jk_ is the j^th^ element of the residual vector **e**_new_ at time k and **ŝ**_jk_ is the standard deviation of the j^th^ variable for the training set of fresh livers.

#### Multiway Partial Least Square Discriminant Analysis (MPLSDA)

While SPE_k_ statistic can evaluate whether a liver is within normal operation range (i.e. how ischemic it is), for livers that are mildly ischemic, it is possible to build on the basis of MPCA to perform a complementary discriminant analysis to classify a given liver either as fresh or ischemic definitively.

To be able to perform online classification of livers as ischemic or fresh, an algorithm that can discriminate different metabolic states from the dynamic metabolite data collected during perfusions is required. Partial least squares or projections to latent structures (PLS) is a regression extension of PCA and is used to connect the information in two blocks of variables, namely the predictor block **X** and response block **Y**. PLS gives a way of predicting the **y**-values from the **x**-values, a generalization of multiple regression, by correlating **X** to **Y** and finding the orthogonal directions that maximize the correlation between **X** and **Y**. An algorithm for Nonlinear Iterative Partial Least Squares (NIPALS) is provided in [30].
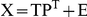


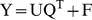
(8)

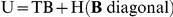
Interrelation gives the predictive formulation for **y**.
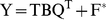
(9)Similar to MPCA, PLS is extended to multi-way PLS (MPLS) for batch processes. MPLS is equivalent to performing PLS where **X** predictor set is the unfolded batch data and **Y** is a 2D matrix of end-of-batch quality variables. The predictor set (**X**) consists of the same 3-D dynamic data collected at each sampling time and analyzed for the 25 metabolite concentrations collected, and unfolded into a I×JK matrix as shown in Figure 1(b). For new data coming from a new perfusion, **x**_new_, MPLS computes the response variables **y**_new_ using the model regression coefficients.

For prediction of the state of a liver as belonging to one of the a priori known classes, partial least squares methodology is named partial least squares discriminant analysis (PLSDA). PLSDA is used to classify observations or independent experiments in the predictor set **X** as belonging to one of several a-priori known classes in **Y** based on their relationship. The response matrix **Y** represents the class membership of each row in **X** by a set of dummy variables. The dummy **Y** matrix consists of *G* columns for *G* classes with 1 s and 0 s such that the *g* ^th^ column is 1 and the other columns are 0 for observations of class *g* (Figure 1(b)). For the analysis of the dynamic metabolite data a multiway PLSDA (MPLSDA) model is fit between **X** matrix and the dummy **Y** matrix (I×G, G = 2 for WI or fresh) and discriminant plane that separates perfusions *i* according to their membership in a certain class is found. When the unfolded new data **x**_new_ is projected onto the MPLSDA model, 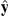 vector is calculated. The predicted class is the numerical maximum of the normalized 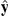 vector.

For the analysis of fresh and ischemic livers, the (I×JK) unfolded predictor matrix (**X**) consists of the metabolite trajectories for the selected ten batches; the response matrix (**Y**) consists of two columns representing the class membership of each of the ten batches. For a fresh liver perfusion batch (class 1), the corresponding row of **Y** is [1, 0], whereas, for a WI liver batch the row is set to [0, 1].

### Multi-sampling cross validation

The predictive power of MPLSDA was evaluated by case-resampling cross validation technique [35], [36]. In this approach, the data set is sampled randomly multiple times to create training and validation data sets. For each sampling, five samples from fresh livers group and five samples from the WI livers are selected randomly and the remaining samples, 5 fresh and 2 WI livers, are used for testing the model. The maximum selection threshold for each batch is set to 2; the maximum selection threshold is the maximum number of times same perfusion can be used in the same model building. The selection threshold is added to avoid the same samples being selected most of the time.

## Results

### MPCA and Outlier Analysis

**Figure 2:**
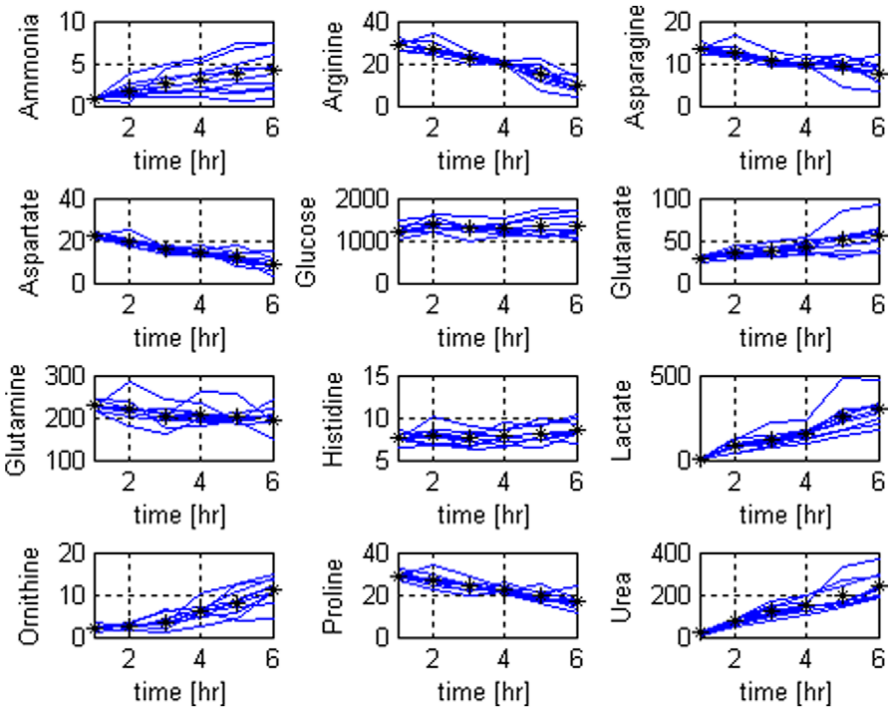
Selected metabolite concentration profiles of fresh livers during 6 hours of normothermic extracorporeal liver perfusion. Blue solid lines show the profiles for the different perfusions of fresh livers and the black stars show the mean values.

MPCA was used to create a model of the variability and the correlation structure of the fresh livers metabolic function during perfusion. The data that is used for the statistical analysis with MPCA is 11×25×6 for fresh livers and 7×25×6 for damaged livers. Several (12 chosen out of 25) metabolite profiles during the perfusions of fresh livers are shown in Figure 2. As it can be observed, the mean variable trajectories change linearly with time indicating that the system was stable during perfusion. An MPCA model with R = 3 components was built after the data is unfolded into a (I×JK) matrix to model the batch-to-batch variation (Figure 1(a)). First three principal components account for more than 65% of the variability in the data.

**Figure 3:**
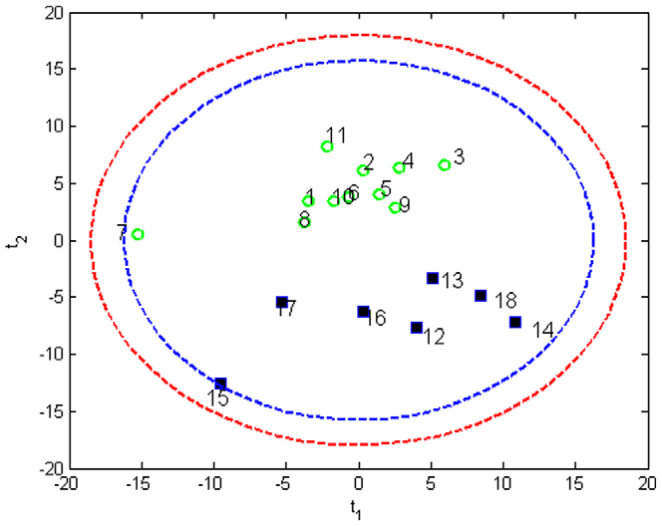
Score bi-plot of all liver samples measured, with 95% and 99% confidence limits. Fresh liver sample 7 and warm ischemic liver sample 15 fall away from the two clusters representing fresh livers and warm ischemic livers. (1–11 fresh samples denoted by green circles, 12–18 warm ischemic samples denoted by squares).

When an MPCA model was built using all the liver samples (n = 18, 11 F and 7 WI) on a 18×25×6 data set, a score biplot (**t**_1_) vs (**t**_2_) reveals the two clusters; one for fresh livers (F) and one for WI livers (Figure 3). Formation of two different clusters is an indication of different metabolic functioning between the two groups. Within the groups each liver has similar metabolic functioning, however, liver samples #7 (F) and #15 (WI) fall away from the two clusters outside the confidence levels and therefore were accepted as outlier samples and are not used further in modeling.

### Index of Ischemia

**Figure 4:**
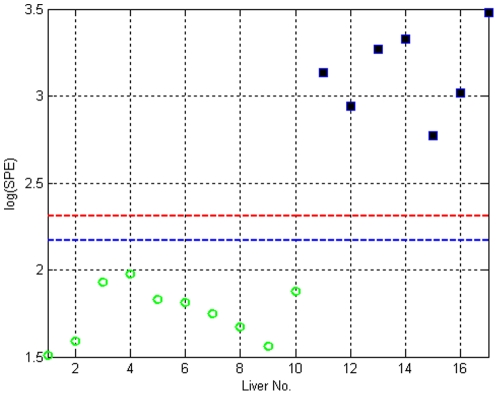
Projection of warm ischemic (WI) liver samples onto the MPCA fresh liver model. Fresh livers are denoted by green circles and are inside the confidence limits (blue: 95%, red: 99%), whereas, the WI livers, denoted by squares are outside the confidence limits.

The MPCA model allows the calculation of SPE statistics for each perfusion (using the residuals in Eq. 3) for fresh and WI perfused livers. Figure 4 depicts the projection of (offline) SPE values for all livers, along with the confidence limits for fresh livers, demonstrating that SPE is clearly able to distinguish between fresh and warm ischemic perfused livers.

**Figure 5:**
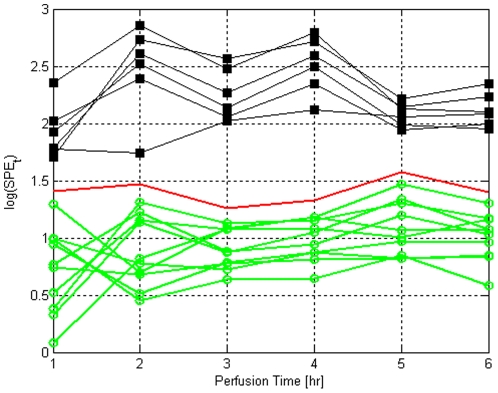
Online SPE statistic for fresh and warm ischemic livers, denoted by green circles and black squares, respectively. The solid line shows the 99% confidence limits.

Perhaps more interesting is the online SPE_k_ statistics which are calculated at each time point using Eq. 5. Figure 5 depicts the time-evolution of the SPE_k_ during perfusion for each liver; again note that the SPE_k_ statistics are online, meaning that they are evaluated at time point *k* using only the data available for that liver until that point. As shown in Figure 5, fresh livers display high variations in SPE_k_ values within the first 2 hours, and stabilize afterwards. In somewhat of a contrast, the changes in SPE_k_ for the WI livers as a function of time are less dramatic. However, interestingly the liver-to-liver variation between SPE_k_ values decreases noticeably in time for ischemic grafts (std (SPE_k_) = [0.2391, 0.3951, 0.2268, 0.2484, 0.1044, 0.1463] for t = 1,…, 6 hrs respectively), and it appears that by the end of perfusion all ischemic livers are in a very similar metabolic state although their initial states are quite different. Still, despite this “standardization”, the SPE_k_ values for the WI livers always remain outside the 99% confidence limits of fresh livers from the beginning of the perfusion till the end, clearly demonstrating that the perfusion-resuscitation of these organs' metabolism does not render them equivalent to that of fresh livers.

As Figure 5 demonstrates, the SPE_k_ statistic quantifies and captures the similarities and differences between ischemic and fresh perfused livers as a group, as well as liver-to-liver variances. It is a single number that can be calculated during perfusion from the metabolite concentrations (see Information S1 for the full equation used for calculations in Figure 5), and compared to existing data to evaluate how fresh or ischemic an organ is. Moreover it is a continuous number; hence, it has the resolution and ability to differentiate between different degrees of ischemic injury. In fact, as displayed in Figure 5, it is capable of catching small metabolic differences, likely small variations due to rat-to-rat differences in metabolism, last time of eating prior to sacrifice, and small differences in the exact ischemia duration. As such, we propose its use as an index of ischemic injury for rat livers. Moreover, as can be observed in Figure 5 for fresh livers, the average confidence limits log(SPE_k_) is ∼1.35. Accordingly, we suggest that log(SPE_k_) as a heuristic limit for ischemia, with values >1.35 indicating ischemia.

Table 2 summarizes the key metabolites that contribute to the SPE_k_. Note that only the variables with higher than the 3σ limits of normal contributions are tabulated. “Normal contributions” are calculated using the fresh liver perfusion profiles. Ornithine (#18), arginine (#5), albumin (#3), and tyrosine (#23) concentrations are significantly different between all fresh and WI livers, although these differences vary from time point to time points. Lactate, glutamine and glutamate are other major contributors to the SPE_k_ values.

**Table 2:** Variable contributions to SPE_k_ statistic.

WI Liver No.	k = 1 hr	k = 2 hr	k = 3 hr	k = 4 hr	k = 5 hr	k = 6 hr
1	Lactate	Ornithine	Arginine, Lactate, Ornithine	Arginine, Glutamate	Albumin, Ornithine	Glutamate, Ornithine, Tyrosine
2	Glutamine	Ornithine	Arginine, Lactate	Arginine, Glutamate	Albumin, Ornithine	Glutamate
3	Lactate	Ornithine	Arginine, Ornithine	Arginine, Glutamate, Ornithine	Albumin, Ornithine	Glutamate, Ornithine, Tyrosine
4	Alanine	Ornithine	Arginine, Lactate, Ornithine	Arginine, Glutamate	Albumin, Ornithine	Ornithine
5	Lactate	Ornithine	Tyrosine	Albumin, Glutamate	Albumin, Ornithine	Glutamate, Tyrosine
6	Aspartate	Ornithine	Arginine, Ornithine	Arginine	Albumin, Ornithine	Glutamate, Ornithine
7	Glutamine	Ornithine	Arginine, Ornithine	Arginine, Glutamate, Ornithine	Ornithine	Glutamate, Tyrosine

**Figure 6:**
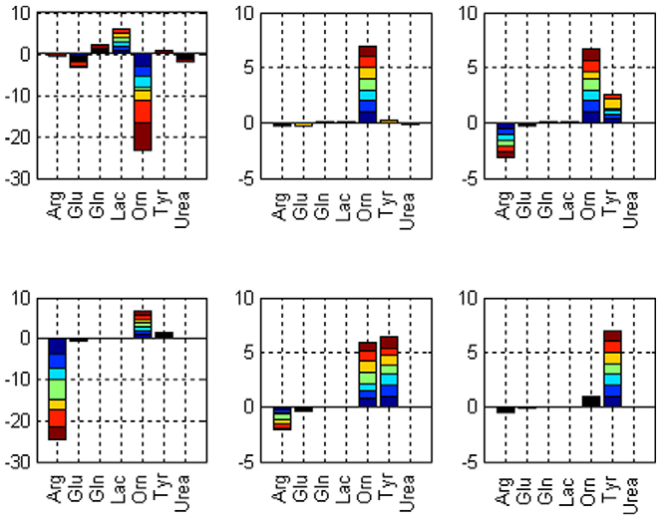
Contributions of selected metabolites to the normalized error vector at each time point for WI livers. Contribution values for each WI liver are stacked in each bar. Six figures correspond to k = 1,…,6 hr time points.

Figure 6 shows the contributions of selected metabolites to the normalized error vector for warm ischemic livers (see Eqns 6 and 7); note that only metabolites with high contributions are shown for clarity. Although albumin is one of the variables with large contribution values, it is not shown in the plot for scalability. Contributions from each perfused graft are stacked with the colors indicating different livers. The advantage of normalized error vector is that it can differentiate between positive and negative variable contributions; a positive value for a particular metabolite indicates that its concentration is higher than the fresh livers. Overall, albumin concentrations in the WI livers are always less than the concentrations for fresh livers. An interesting note is that ornithine levels start negative, and switch to positive at 2 hrs and on. Arginine concentration starts decreasing after the third hour and drops to a minimum at time = 4 hr. Tyrosine levels increase towards the end of the perfusion.

### Classification

While SPE_k_ provides a continuous index to evaluate a given liver, the log(SPE_k_)>1.35 criterion suggested above is a heuristic. The relatively wide margin of SPE_k_ values (Figure 5) can make classification problematic in the circumstance that a liver is equally different from ischemic and fresh livers. Accordingly, to complement the index we built and employed an MPLS-DA model to solve this corollary classification problem, and evaluated its accuracy by multi-sampling cross validation.

**Figure 7:**
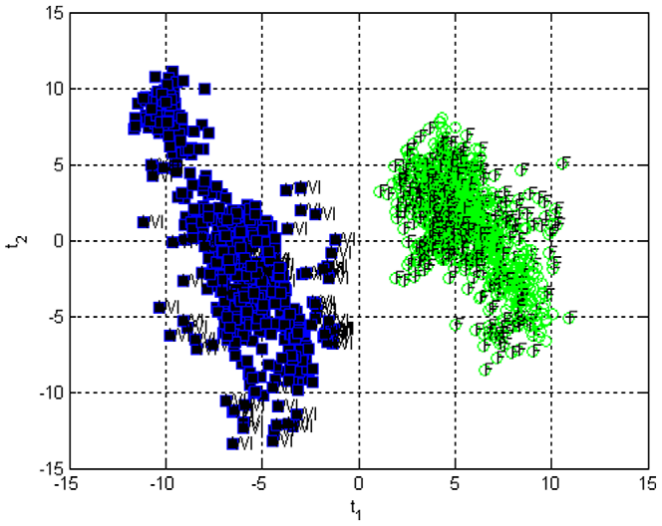
Offline classification of fresh (F) and warm ischemic (WI) liver samples using case resampling cross-validation. During cross-validations some of the liver samples were left out to be used in model testing. These samples are projected onto the PLSDA model and they are denoted as WI (squared) and F (circles). All of the test samples for all 100 models were clustered correctly.

An MPLSDA model with five fresh liver samples and five warm ischemic liver samples using three principal components was developed and found to explain more than 60% of the variation in **X** and 99% variation in **Y**. The results of offline classification using 100 runs are illustrated in Figure 7. The perfusions used in the modeling are plotted along with the projections. The perfusions that were not used in model building (five fresh livers and two WI livers in each run) were used to evaluate the accuracy of the model. As it can be observed, the model correctly classifies all of the liver samples in all 100 runs as fresh or warm ischemic (accuracy, specificity and sensitivity = 1). The test was repeated with 50 and 500 runs as well and the results did not change (results not shown).

**Figure 8:**
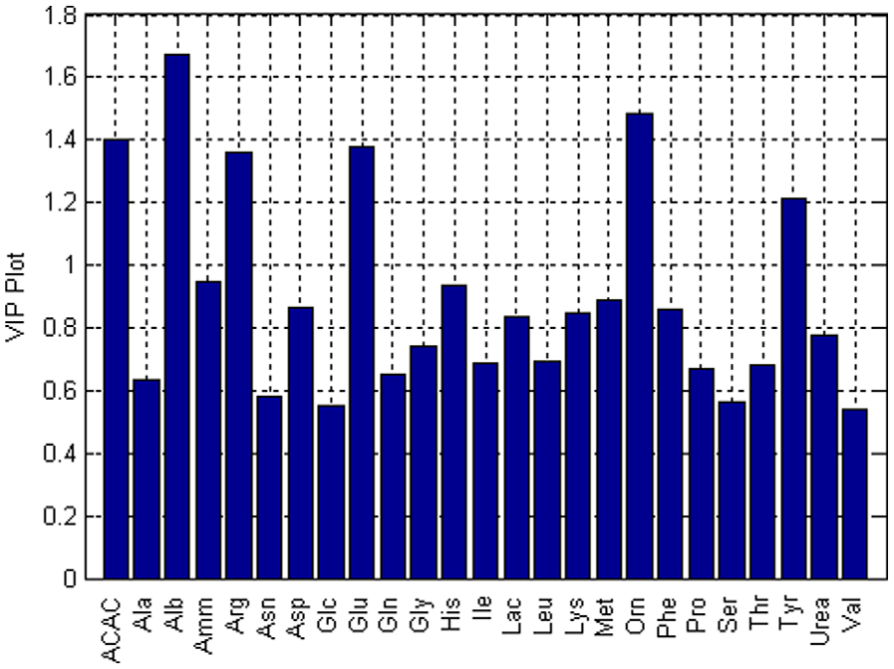
Important variables found in MPLSDA. The most important variables that mark the differentiation of warm ischemic livers from fresh perfused livers have VIP values greater than 1.

Figure 8 shows the most important variables in determination of class membership for each liver sample. Variable importance in the projection (VIP) was calculated by summing the squared PLS weights over all dimensions. The variables with VIP >1 are effective on the response **Y** [16]. The most important variables that separate WI livers from fresh livers are albumin (#3), arginine (#5), glutamate (#9), ornithine (#18), and tyrosine (#23), similar to the analysis before with SPE_k_ except acetoacetate is also chosen for MPLSDA.

With the ability of MPLSDA confirmed in offline mode, an on-line implementation was then tested. Similar to the online ischemia index, the basic idea is to be able to classify a perfused graft as healthy or ischemic during perfusion so that a decision can be made about the organ quality. Such a determination will ultimately be necessary to identify the need for further treatments, and to build a decision criterion for fitness of the graft for transplantation.

**Figure 9:**
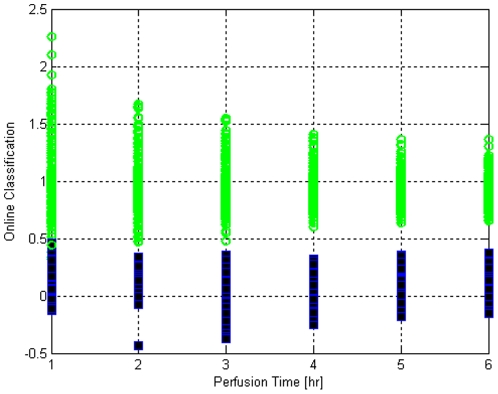
Online classification for fresh and warm ischemic metabolite profiles using case resampling cross validation. Fresh liver samples and warm ischemic livers are represented by circles (>0.5) and squares (<0.5), respectively. Results shown for 50 runs with 10 random livers selected equally from fresh and WI samples.

The prediction power of MPLS was also tested with case-resampling cross-validation, results of classifications are given in Table 3 for 100 resampling runs. Via online MPLS, end of perfusion quality of a liver can be estimated hourly as the perfusion is progressing. End-of-perfusion quality estimates for different perfusions at each hour are shown in Figure 9. The perfusions that are not selected via case-resampling are used for testing the MPLS model and are shown in the figure. Fresh perfusions are denoted by green circles, whereas, the WI liver perfusions are denoted by black-filled squares. In order for a perfusion to be classified as a fresh liver perfusion, the quality estimate should be greater than 0.5, else it is classified as an ischemic liver perfusion. Most of the misclassifications occur during the first two hours and after the third hour usually there are no misclassifications. Detailed specificity and sensitivity values for each time point are displayed in Table 4. As also displayed in Table 4, to ensure that the specificity and sensitivity evaluations are not dependent on the cross validation parameters, analysis was repeated with three different numbers of resampling runs; indeed the variation due to number of replicates is minimal. Moreover, specificity and sensitivity are always above 0.98 which is excellent for online classification. It is notable that misclassifications during online classification are more common in the first 3 hrs, although the difference is essentially negligible.

**Table 3:** Sensitivity and specificity analyses for online quality prediction via MPLS for 100 cross-validation runs.

	WI (Actual)	Fresh (Actual)
Classified as WI	599	10
Classified as Fresh	1	2990

**Table 4:** Sensitivity and specificity analyses for online quality prediction using MPLS for 50, 100 and 1000 runs.

Criterion	k = 1 hr	k = 2 hr	k = 3 hr	k = 4 hr	k = 5 hr	k = 6 hr
Sensitivity (50 runs)	1	1	0.980	1	1	1
Specificity (50 runs)	0.988	0.984	0.992	1	1	1
Sensitivity (100 runs)	1	1	0.990	1	1	1
Specificity (100 runs)	0.986	0.9940	1	1	1	1
Sensitivity (1000 runs)	0.995	1	0.997	1	0.999	1
Specificity (1000 runs)	0.984	0.990	0.997	1	1	1

## Discussion

In this work we introduce statistical process monitoring methodologies for evaluation of ischemic injury of cadaveric livers via their metabolic functions during extracorporeal perfusion. We focused specifically on hepatic metabolism because of the large integrated body of data with which to systemically gauge organ functional status, compared to the more traditionally employed singular tests of cellular injury, such as liver enzyme release (ALT & AST) [37], oxygen uptake, [12] or ATP [38]. Our goal was to test the hypothesis that liver metabolic performance can be employed to evaluate the degree of ischemic injury cadaveric organs have sustained, with the ultimate goal of developing a metabolic feedback control scheme for optimizing organ viability and transplantability on-line.

The multivariate analysis performed demonstrated that fresh and ischemic rat livers display clearly distinguishable metabolic activities. Further, it was possible to distill these differences into a single metric, the multivariate SPE_k_ statistic, which we; therefore conclude that the SPE_k_ statistic can be employed as an accurate and continuous index of ischemic injury for perfused rat livers. Moreover, it was demonstrated that this statistic can be computed online during perfusion, and hence can be used in the future to build a more sophisticated feedback-control scheme for regulation of perfusate supplements. Based on our data, a cutoff value for SPE_k_ was determined to be used as a heuristic limit of ischemia. This analysis can be further expanded to explore the correlations between metabolic function and traditional tests of cellular injury, and translated to human livers for a clinically applicable index of ischemia.

We also performed a complementary discriminant analysis to classify new livers with unknown conditions as either fresh or ischemic. Whereas the SPE_k_ creates a continuous index that is ideal for utilization in on-line regulation/optimization of organ function and viability, discriminant analysis approach enables a final determination to be made if the organ is ready for transplantation or not. Specificity and sensitivity of the MPLSDA model we created were both >0.98, the accuracy of which was tested through multi-sampling cross validation. As in the index of ischemia/SPE_k_, both offline and online analysis was performed and demonstrated to be successful in discriminating new perfusion data. It was noted that online MPLSDA had a few misses in the first 2 hours, and that these errors disappeared completely at t = 3 hr and on. Two possible explanations here are i) fully sufficient data for discriminant analysis becomes available only after three time points of evaluation, or ii) the variability among fresh livers diminishes after the second hour when their functions stabilize, hence allowing easier separation of fresh livers from ischemic livers. If the latter case is valid, this *might* indicate that all the perfused livers have recovered from hypoxia sufficiently to perform measured metabolic functions, and therefore perfusion has achieved its goal at t = 3 hrs and further perfusion is not necessary. In fact, the ability to identify when the liver is ready for transplantation would be very valuable for clinical utility of perfusion systems as such a measure is currently not available. However, it is important to note though that while the models proposed and developed in this work can identify the degree of ischemia, they cannot be used for evaluation of transplantability since all the livers perfused were successfully transplanted. However, this model can be the basis for determining transplantability if data from extended ischemic organs where recipients fail is included in the data. Testing by further transplantation at shorter perfusion durations is necessary for development of such an index.

The multivariate SPE_k_ statistic identified arginine, ornithine, albumin, tyrosine, lactate, glutamate, with the addition on acetoacetate in the MPLSDA model, as key metabolites that distinguished fresh from ischemic livers. The majority of these metabolites are directly associated with ischemia-induced pathway alterations. Arginine, for instance, is a precursor to both the vasodilator nitric oxide (NO) and ornithine. The balance between arginases (producing ornithine) and nitric oxide synthases (producing NO and citrulline) are dramatically altered during hypoxia [39], [40], [41]. This may account for the significantly higher rate of arginine depletion observed in WI livers compared to Fresh livers (Figure 6), with a concomitant increase in ornithine production (Figure 6). Tyrosine, normally catalyzed in the liver to acetoacetate and fumarate, acts a precursor to nitrotyrosine, a derivative of altered NO metabolism, the increased presence of which may be associated with suboptimal levels of arginine [42]. Further investigations to elucidate the role of arginine in hepatic microvasculature preservation, and verification of the dominant by-products of tyrosine metabolism during perfusion of Fresh and WI livers, will enable optimal priming of the perfusate to minimize reperfusion injury. Albumin is a negative acute phase protein such that its production decreases when hepatocytes are stressed. Interestingly, a known trigger that reduces albumin synthesis is increased intracellular NO as a measure of reducing energy expenditure during hypoxia [43]; the effects may last for several hours [44]. From our findings, albumin synthesis in WI livers remained flat for the duration of perfusion, while Fresh livers began to recover by t = 2 hours. Lactate is a by-product of anaerobic metabolism; while cells will secrete lactate into a perfusate devoid of any lactate [45], lactate production exceeding this rate by WI livers in the first hour of perfusion suggests a response to their inadequate oxygen supply (Figure 6). Glutamate production as a marker of amino acid transamination increases throughout perfusion of Fresh livers, but is constant in WI livers. This would be consistent with the overall reduction in protein synthesis by WI livers, requiring less amino acid degradation, or an upregulation of the urea cycle by these livers, driven by their increased arginine consumption.

The analyses performed in this work all confirm that a metabolic index of ischemic injury is a feasible idea for evaluation of perfused ischemic livers, and such a measure would be of significant use in utilization of DCD livers for transplantation. Similar data for human livers is not available; however, in principle, the proposed methodology can similarly be applied to human livers in a clinical setting. This study demonstrates the power of SPM methodologies in achieving this goal; however, further work is needed to reach an index of transplantability. The data gathered here can also be used for more sophisticated metabolic analyses which reveal more details of the cellular function [46], [47], [48].

## Supporting Information

Information S1 **Online MPCA, MPLS and calculation of index of ischemia for new perfusions.**
(DOC)
