# Supplementary material for: A Metabolic Index of Ischemic Injury for Perfusion-Recovery of Cadaveric Rat Livers
Source: PLoS One. 2011 Dec 14;6(12):e28518. doi: 10.1371/journal.pone.0028518 (PMC3237452; doi:10.1371/journal.pone.0028518)
Supplement: Information S1 — Online MPCA, MPLS and calculation of index of ischemia for new perfusions. (DOC) [file pone.0028518.s001.doc]

**SUPPORTING INFORMATION S1**

**Online MPCA, MPLS and calculation of index of ischemia for new perfusions**

For both MPCA and MPLS applications, first a model is formed using I batches (X is of size I x JK). When a new perfusion is being monitored online, new data is projected onto the model as it becomes available during perfusion. An online application uses the already known observations until time interval k is denoted by x_new,kJ_ (1 x kJ).

After the model is built, new data is projected onto the model using the model parameters. Online MPCA scores are calculated using $\mathbf{t}_{k}=\mathbf{x}_{new,jk}\mathbf{P}_{jk}\left(\mathbf{P}^{T}\mathbf{P}\right)^{-1}$. $\mathbf{P}_{jk}=(kJ\times R)$are the model loadings until time interval k and define the new model plane based on the R eigenvectors that correspond to R largest variance directions. The **P** matrix used in the MPCA rat model of fresh livers with 3 PCs is given in Figure S1. Residuals are then calculated using $\mathbf{e}_{new}=\mathbf{x}_{new}-\mathbf{t}_{k}\mathbf{P}_{k}^{T}$, and SPE is calculated at time k using SPE = **e**_new_**e**_new_^T^.

Similarly, online MPLS is performed at time k using the model parameters **B**, **Q**, **P**, and **W**. New online scores are calculated using $\hat{\mathbf{t}}=\mathbf{x}_{new,k}\mathbf{W}_{1:kJ,R}\left(\mathbf{P}^{T}\mathbf{W}\right)^{-1}$. Once the scores are available **y**, response or quality variables, is predicted using scores and model parameters **B**, **Q** as . Residuals and the SPE statistics for MPLS are calculated similar to MPCA.

**Algorithm for the calculation of ischemia index for new perfusions.** User-defined measurement vector **x**_new,jk_ (1 x kJ) contains the measurements of J variables until time k of the new batch **x**_new_ (1 x JK). At each time interval k, only the measurements until that time point are available. **P** is given in Figure S1 for the dataset used in this study. **P**_jk_ (kJ x R) is the part of **P** (JK x R) that contains the variable loadings until time interval k.

At time k, plug in the available measurement vector **x**_new,jk_ (1 x kJ) into the following formulation using the provided P matrix to calculate the index of ischemia for a new liver.

1. Calculate $\mathbf{t}_{k}=\mathbf{x}_{new,jk}\mathbf{P}_{jk}(\mathbf{P}^{T}\mathbf{P})^{-1}$
2. Residuals are $\mathbf{e}_{k}=\mathbf{x}_{new,jk}-\mathbf{t}_{k}\mathbf{P}_{jk}^{T}$
3. Ischemia index at time k is $\log(SPE_{k})=\log(\mathbf{e}_{k}\mathbf{e}_{k}^{T})$, and the liver is ischemic if log(SPE_k_) > 1.35.


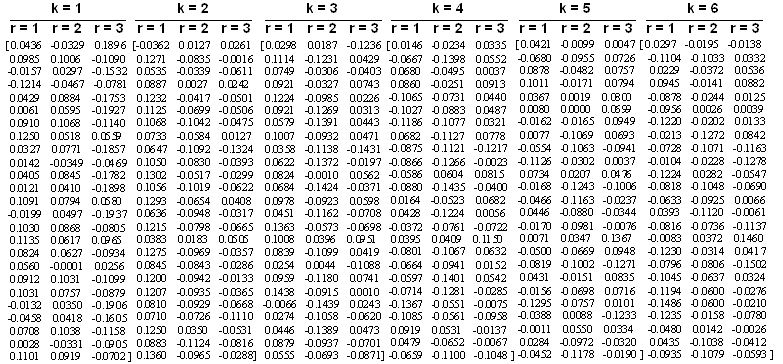


Figure S1: A sample P matrix (25 x 3 x 6) for the MPCA model. The columns correspond to three principal components (R = 3) for k = 6 sampling times and the rows show the weights for the variables (N = 25).
